# Virtual reality-based monitoring test for MCI: A multicenter feasibility study

**DOI:** 10.3389/fpsyt.2022.1057513

**Published:** 2023-01-18

**Authors:** Sooah Jang, Sun-Woo Choi, Sang Joon Son, Jooyoung Oh, Junghee Ha, Woo Jung Kim, Hyun Woong Roh, Keun You Kim, San Lee, Eunjin Jung, Woojin Cha, Heonjoo Chae, Suzi Kang, Ji Hye Kwon, In-Young Kim, Ju-Yeal Lee, Hyun Kyung Shin, Jin Sun Ryu, Ryunsup Ahn, Chang Hyung Hong, Jeong-Ho Seok

**Affiliations:** ^1^Research Institute of Minds.AI, Co., Ltd., Seoul, Republic of Korea; ^2^Department of Psychiatry, Ajou University, Suwon, Gyeonggi-do, Republic of Korea; ^3^Department of Psychiatry, Gangnam Severance Hospital, Yonsei University College of Medicine, Seoul, Republic of Korea; ^4^Institute of Behavioral Sciences in Medicine, Yonsei University College of Medicine, Seoul, Republic of Korea; ^5^Department of Psychiatry, Severance Hospital, Yonsei University College of Medicine, Seoul, Republic of Korea; ^6^Department of Psychiatry, Yongin Severance Hospital, Yonsei University College of Medicine, Yongin, Gyeonggi-do, Republic of Korea; ^7^FNIKorea Co., Ltd., Gwacheon, Gyeonggi-do, Republic of Korea

**Keywords:** virtual reality, mild cognitive impairment, screening, cognitive assessment, MOCA, DHEA

## Abstract

**Objectives:**

As the significance of the early diagnosis of mild cognitive impairment (MCI) has emerged, it is necessary to develop corresponding screening tools with high ecological validity and feasible biomarkers. Virtual reality (VR)-based cognitive assessment program, which is close to the daily life of the older adults, can be suitable screening tools for MCI with ecological validity and accessibility. Meanwhile, dehydroepiandrosterone (DHEA) has been observed at a low concentration in the older adults with dementia or cognitive decline, indicating its potential as a biomarker of MCI. This study aimed to determine the efficacy and usability of a VR cognitive assessment program and salivary DHEA for screening MCI.

**Methods:**

The VR cognitive assessment program and the traditional Montreal Cognitive Assessment (MOCA) test were performed on 12 patients with MCI and 108 healthy older adults. The VR program operates in a situation of caring for a grandchild, and evaluates the memory, attention, visuospatial, and executive functions. An analysis of covariance (ANCOVA), a partial correlation analysis, and receiving operating characteristic (ROC) curve analysis were conducted for statistical analysis.

**Results:**

According to the ANCOVA, no significant difference in MOCA scores was found between the normal and MCI groups (*F* = 2.36, *p* = 0.127). However, the VR total score of the MCI group was significantly lower than that of the normal group (*F* = 8.674, *p* = 0.004). There was a significant correlation between the MOCA and VR scores in the total and matched subdomain scores. The ROC curve analysis also showed a larger area under the curve (AUC) for the VR test (0.765) than for the MOCA test (0.598), and the sensitivity and specificity of the VR program were 0.833 and 0.722, respectively. Salivary DHEA was correlated with VR total (*R*^2^ = 0.082, *p* = 0.01) and attention scores (*R*^2^ = 0.086, *p* = 0.009).

**Conclusion:**

The VR cognitive test was as effective as the traditional MOCA test in the MCI classification and safe enough for older adults to perform, indicating its potential as a diagnostic tool. It has also been shown that salivary DHEA can be used as a biomarker for MCI.

## 1. Introduction

As the older adult population has increased, the burden of cognitive impairment has increased as well. There is a growing interest in the diagnosis of mild cognitive impairment (MCI) and early intervention for the prevention of conversion to dementia. MCI is defined as the pre-dementia stage, in which cognitive impairment has not yet caused major problems in one’s daily function (1). A proper screening tool is important as 6–15% of MCI cases convert to dementia each year, and early diagnosis can modify the conversion predictors and allow patients and families to improve or maintain their quality of life (2–4). MCI has been defined as a broad concept; however, the concept defined by Petersen has been presented as a standard (5). Several neuropsychological tests, including the Mini-Mental State Examination (MMSE) and Montreal Cognitive Assessment (MOCA), have been used to detect cognitive impairments.

However, there has been a gap between the results of these classical tests and the actual difficulties faced by patients and families in real life. In other words, these tests do not capture the cognitive dysfunction associated with problems that are practically revealed in the daily lives of cognitively impaired patients, indicating a lack of ecological validity (6). Traditional tests also have limitations, such as the uncomfortable experience associated with taking them and the necessity of professionals (7). Therefore, there is a need for a more objective MCI screening test with ecological validity or the use of biomarkers.

Virtual reality (VR) technology, which can provide computer-generated 3D environments mimicking the real world, has been applied as an effective tool with ecological validity in several fields (8). Several studies have suggested that neuropsychological assessment using VR is useful in detecting cognitive decline in older adults, as the feasibility and accessibility (9–13) VR can provide more interactions with the subjects compared to paper-based tests, and it can be easily tailored to control the level of stimulation as desired by the examiner (10). VR games for cognitive evaluation or cognitive training have been developed for older adults (9, 10, 13). These VR games have been reported to increase the ecological validity by allowing older adults to play in the background of everyday life. In addition, when a paper-based test is performed, bias may occur depending on the educational level or anxiety of a patient. Test dependent bias can be minimized in the form of a game (9).

Various VR tests have been developed to screen MCI and dementia, and their validity and reliability have been confirmed (14). Recently developed VR tests for MCI diagnosis are performed in one cognitive domain, such as spatial and episodic memory, and most of the tests include one or two tasks (14). These tests have proven discriminative potential; however, the sensitivity of a single domain is lower than that of tests that measure both memory and executive functions (14). In addition, previous studies have shown that the evaluation of attention, delayed recall, and visuospatial functions, such as Rey-Osterrieth compex figure test, increase the discriminating power (7). Therefore, the development of a more sensitive VR test is required to evaluate multiple cognitive domains through various tasks.

In addition, verisimilitude, the degree to which the cognitive demands of a test resemble the cognitive demands in the everyday environment, must be taken into account for a higher ecological validity (15). Therefore, it is important to provide a VR environment that is closely related to the lives of the older adults. Existing MCI-discrimination tests using VR technologies are often reminiscent of video games, such as finding the road (16), driving (17), finding a treasure in a maze (11), and performing archaeological missions in museums (18). Currently, only a few VR programs offer environments related to daily life, such as supermarkets, looking for objects in the house, withdrawing money, or riding a bus (12, 13, 19). Therefore, we believe that the development of VR tests based on daily life is necessary to increase the ecological validity with an authentic approach.

Regarding biomarkers, imaging studies such as magnetic resonance imaging and positron emission tomography or detection of amyloid-beta and tau proteins in cerebrospinal fluid (CSF) have been candidates for effective MCI screening (20). However, they are not suitable as screening tools due to their high cost and inaccessibleness of imaging equipment or invasiveness of spinal tapping. Recently, the link between metabolism and Alzheimer’s disease (AD) has been explored, and the cholesterol pathway has been shown to be linked to biomarkers of cognitive decline (21, 22).

Dehydroepiandrosterone (DHEA), a neuroactive steroid, is known to affect synaptic connectivity and neuronal differentiation, and has an important effect on cognitive decline and AD development (23). A decline in DHEA levels was found in the brains of patients with AD, and a negative correlation between DHEA and AD-related peptides, such as amyloid beta and phosphorylated tau, was observed in the brain. Since DHEA is a factor that affects the occurrence of AD, it has the potential to be a biomarker of MCI, which is a stage of transition (24, 25). If DHEA can be obtained from an easily accessible body fluid, such as saliva, rather than from the CSF or blood, it could have the potential to be a powerful screening tool.

Given these premises, the present study was performed with three aims. First, we investigated the validity of the virtual reality-based MCI monitoring (VARABOM) test using 12 tasks on multiple cognitive subdomains to discriminate between MCI and healthy controls in comparison with the traditional MOCA test. Second, the usability of the VR cognitive test was evaluated in a cohort of patients with MCI and healthy controls (HCs). Third, salivary DHEA levels were analyzed to determine their relationship with MCI and their potential as a screening biomarker.

## 2. Materials and methods

### 2.1. Participants

This study was conducted based on the results of a VR cognitive test and DHEA hormone analysis from the VARABOM project, which involved the proactive collection of cognitive function, psychological status, and psychophysiological signals for older adult mental health monitoring in the community. The VARABOM project is an ongoing program that was launched to develop mental health evaluation and treatment programs for older adults. The psychiatric department of the four general hospitals and two community mental health centers for older adults are involved together. Data recruitment began in August 2021, and 150 samples were collected by December 2021. Out of a total of 150 samples, 120 people who underwent VR assessment were included in the final analysis of this study. Thirty people who initially used the program when there was an error in the VR program itself and experienced interruptions in use were excluded from the analysis. There were no cases where patients stopped using VR because they found it difficult to use VR on their own. The participants were recruited through posts from the outpatient clinic of a university hospital and community mental health centers for older adults. The participating institutions were Severance Hospital, Gangnam Severance Hospital, Yongin Severance Hospital, Ajou University Hospital, Seodaemun-Gu Dementia Reassuring Center, and Suwon Geriatric Mental Health Center. The selection criteria of VARABOM project were those aged 60 years or older who consented to and agreed with the purpose of the study. Volunteers who were recruited from the post were referred to the clinic for baseline demographic evaluation, psychological questionnaire, MOCA test, VR test, and heart rate variability (HRV) test by researchers in our team. The results of the HRV test were not used in the current study. After that, the saliva collection kit was distributed, and participants were guided to collect hormones on the evening of the day and the morning of the next day. The inclusion criteria of the MCI group were (1) subjective cognitive decline and (2) GDS score 3. The criteria for normal group were (1) GDS score 1 or 2 and (2) absent of previous MCI or dementia diagnosis. The exclusion criteria were as follows: (1) severe physical/neurological disease; (2) history of cardiovascular disease; (3) current use of steroid hormones, hormonal drugs, or herbal medicines; (4) history of schizophrenia or substance alcohol use disorders; and (5) difficulty in communicating properly with researchers, or difficulty in getting help from guardians. People with depression, anxiety, and sleep disorder history were included in our analysis, and there was no difference in the proportion of psychiatric disease history by group (Table 1). Written informed consent was obtained from all participants. This study was approved by the Institutional Review Boards of Severance Hospital (No.1-2021-0014), Gangnam Severance Hospital (No. 3-2020-0533), Yongin Severance Hospital (No. 9-2020-0137), and Ajou University (No. AJIRB-MED-DE2-20-470).

**TABLE 1:** Demographic and clinical characteristics of normal and MCI groups.

	Normal (*n* = 108)	MCI (*n* = 12)	*t*/χ^2^	*p*
Age, mean (SD)	72.3 (6.2)	75.8 (7.7)	-1.835	0.069
Sex, *n* (%)			0.017	0.896
Male 0	34 (31.5%)	4 (48%)		
Female 0	74 (68.5%)	8 (52%)		
Education year, *n* (%)			4.477	0.345
0	2 (1.9%)	1 (8.3%)		
1–6	16 (14.8%)	1 (8.3%)		
7–9	14 (13%)	3 (25%)		
10–12	38 (35.2%)	5 (41.7%)		
≥12	38 (35.3%)	2 (16.6%)		
Marriage, *n* (%)			0.728	0.948
Unmarried	3 (2.8%)	0 (0%)		
Married	74 (68.5%)	8 (66.7%)		
Cohabit	2 (1.9%)	0 (0%)		
Divorced	7 (6.5%)	1 (8.3%)		
Bereaved	22 (20.4%)	3 (25%)		
Occupation, *n* (%)			0.897	0.947
Employed	23 (21.3%)	4 (33.3%)		
Unemployed	85 (78.7%)	8 (66.7%)		
Psychiatric disease history, *n* (%)			0.144	0.705
Yes	39 (56.5%)	5 (41.7%)		
No	69 (63.9%)	7 (58.3%)		
Depressive disorder history, *n* (%)			0.745	0.388
Yes	24 (22.2%)	4 (33.3%)		
No	84 (77.8%	8 (66.7%)		
Current psychiatric medication, *n* (%)			1.263	0.261
Yes	21 (19.4%)	4 (33.3%)		
No	87 (80.6%)	8 (66.7%)		
Current cognitive medication			3.333	0.068
Yes	9 (8.3%)	3 (25%)		
No	99 (91.7%)	9 (75%)		
SGDS-K*, mean (SD)	4.26 (4)	5.09 (3.53)	-0.657	0.513
PROVE-DS**, mean (SD)	15.77 (11.9)	14.5 (12.47)	0.347	0.729
PROVE-SR**, mean (SD)	2.98 (2.14)	2.75 (2.56)	0.349	0.728
BS4MI-elderly, *n* (%)			4.107	0.128
Green (normal)	49 (45.4%)	3 (25%)		
Yellow (risk)	49 (45.4%)	9 (75%)		
Red (disorder)	10 (9.3%)	0 (0%)		
Computer use experience, *n* (%)			0.768	0.381
Yes	68 (63%)	6 (50%)		
No	40 (37%)	6 (50%)		
Smartphone use experience, *n* (%)			1.034	0.309
Yes	105 (97.2%)	11 (91.7%)		
No	3 (2.8%)	1 (8.3%)		
DHEAsum (nmol/L), mean (SD)	5.53 (4.01)	5.6 (4.4)	0.241	0.624

*The number of participants who implemented the scale was 102 in the normal group and 11 in the MCI group.

**The number of people who implemented the scale were 107 in the normal group and 12 in the MCI group.

MCI, mild cognitive impairment; SD, standard deviation; SGDS-K, short form of the Korean Geriatric Depression Scale; PROVE, PROtective and Vulnerable factors battEry questionnaire; DS, depressive symptomatology; SR, suicide risk; BS4MI-elderly, brief screening for four mental illnesses in the elderly; DHEA, dehydroepiandrosterone.

### 2.2. Assessment and measurements

#### 2.2.1. Sociodemographic characteristics

The subjects were investigated for their history of depression, psychiatric drug use, and drug use history, which could affect their cognitive function. In addition, age, gender, years of education, current occupational status, and marital status were also examined. The experience of using digital devices was surveyed according to their computer and smartphone use.

#### 2.2.2. MCI diagnosis – Global Deterioration Scale (GDS)

In this study, the MCI group division was not achieved through detailed criteria, such as clinical trial, based on the neuropsychological test. Instead, the Global Deterioration Scale (GDS) was used to discriminate MCI groups from the normal group (26). The GDS is a brief clinical rating of dementia severity that requires a clinician to rate the severity on a scale from 1 (asymptomatic) to 7 (late dementia). This scale emphasizes memory function and activities of daily living. Stage 1 is asymptomatic, and stage 2 is age-related cognitive decline. Stage 3 has been classified as MCI, which reliably identifies individuals who proceed to subsequent cognitive decline and conversion to probable AD (27–29). Stage 3 of GDS has been defined as the period when objective cognitive decline is observed along with subjective symptoms, which is consistent with the definition of MCI defined by Peterson (26, 29). Accordingly, participants in stage 3 were classified into the MCI group, and those in stages 1 and 2 were classified into the normal group. Skilled clinicians from each institution assessed the patients’ GDS scores.

#### 2.2.3. Cognitive assessment

##### 2.2.3.1. Montreal Cognitive Assessment-Korean version (MOCA-K)

MOCA is a tool that can distinguish MCI from normal aging or AD by simply screening the cognitive function, and has high sensitivity and specificity (30). Visuospatial/executive function, naming, attention, language, abstraction, delayed recall, and orientation were evaluated as subdomains, and 30 points were scored using the Korean version (31). Subjects with an education level of 6 years or less were added 1 point, and the cut-off score for MCI was less than 23 points. The cutoff point by age and education level is shown in Kwak’s study (32).

##### 2.2.3.2. Virtual reality-based MCI monitoring (VARABOM) test

**FIGURE 1:**
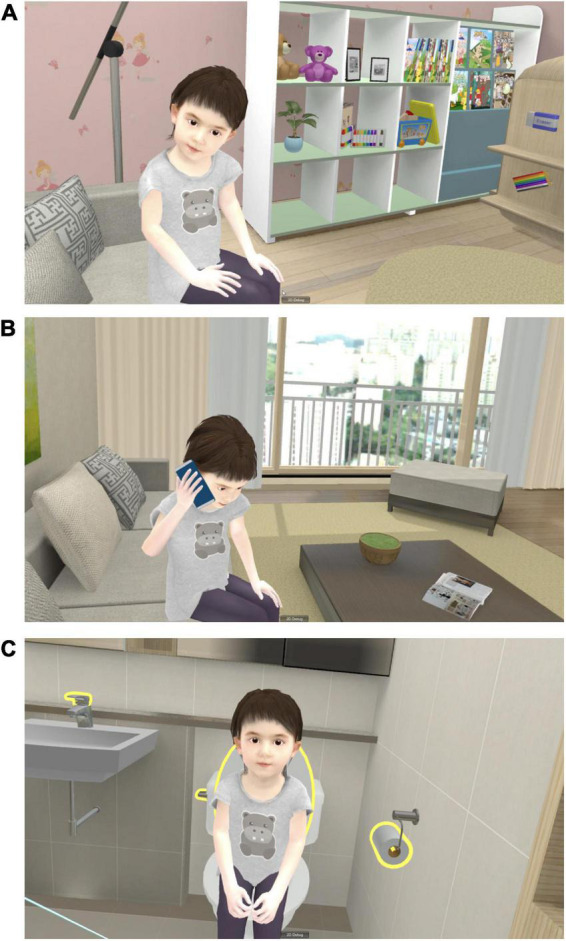
Screenshots of VARABOM test. **(A)** In the “preparation recall” mission, the grandchild asks to find the preparation which was told before. **(B)** In the “phone call” mission, the grandchild asks to call her mother and tells the phone number. **(C)** In the “toilet use” mission, the grandchild asks to help her use toilet.

The VARABOM test was developed using the Unity Editor 2019.3 (FNI Korea, Gyeonggi-do, Republic of Korea; MindsAI, Seoul, Republic of Korea). The program was driven through a kiosk (Elivision, Incheon, Republic of Korea) with Intel i7-8700, 16GB RAM, Nvidia GTX 1660 PC, and the Samsung head mount display (HMD) Odyssey. Kiosks were installed at each clinic. The participants were instructed to sit in a kiosk-tailored chair, wear the HMD equipment, and were informed to select answers using a controller. This was achieved by experiencing the immersive program through the HMD; however, the patients were not allowed to experience the movement directly within the program, which minimized motion sickness. The VR screen viewed by the subjects consisted of the scenes shown in Figure 1.

The VARABOM test is designed to evaluate the cognitive function of memory, attention, visuospatial, and executive functions in accordance with the situation in the context of the older adults taking care of their granddaughters. The tasks were related to real-life activities based on the principles of memory registration, recall, word syllable backward span, digit span, and shape contouring used in classic neuropsychological tests. This program was created by incorporating previously verified tests in everyday life environment as much as possible, with the aim of enhancing ecological validity in cognitive evaluation. A previous study showed feasibility of VARABOM test in assessing cognitive deficits in MCI (33). The entire program consisted of 12 tasks, including six indoor scenarios and six outdoor scenarios near the house.

Detailed contents of the tasks according to each subdomain are listed in Table 2. Memory was evaluated by (1) listening to granddaughter’s list of supplies and checking immediate memory, (2) recalling the supplies after a few minutes, (3) remembering the rides she rode, (4) finding the way to her friend’s house, and (5) remembering the shape of her hat. Attention was evaluated by (1) listening to the phone number and making a phone call, (2) identifying the word syllable backward, and (3) helping the granddaughter choose desired rides in order in the playground. Visuospatial function was measured by (1) looking at cards with complex shapes and finding the same shape, and (2) matching the same flower picture. Executive function was evaluated by (1) helping the children to use the toilet, and (2) helping children with snack time. When the participants gave a wrong answer, it might have proceeded immediately to the next task, or a total of two or three opportunities were given depending on the task. The scores of each task were calculated individually in a determined manner, and they were added without weight when calculating the scores for each area and the entire area. The number of correct answers was considered for each task, and the number of additional attempts was added in reverse for some tasks. Detailed scoring principles are described in Table 2. For each domain, 21 points for memory, 14 points for attention, 6 points for executive function, and 9 points for visuospatial function were set as perfect scores, with a total score of 50 points.

**TABLE 2:** Overview of virtual reality cognitive function test program.

Cognitive subdomain	Task	Contents	Score	Scoring principle
Memory	Preparation	Listen to what supplies should be packed, and register the memory	6	Number of hits on the first attempt (3 points in total) + (3-failure count)
	Preparation recall	Choose the supplies that were remembered before	5	Number of hits on the first attempt (3 points in total) + (2-failure count)
	Playground recall	Remember the playground rides that were played on before	3	Number of hits on the first attempt (3 points in total)
	Path finding	Remember the way to a friend’s house, and find the way again	3	1 point for each hit
	Hat finding	Remember the shape of the hat, and find it	4	1 point for each hit
Attention	Phone call	Listen to the phone numbers, and dial the numbers	4	Points according to the number of hits on the first attempt; 7–8 hits: 4 points 5–6 hits: 3 points 3–4 hits: 2 points 1–2 hits: 1 point 0 hit: 0 point
	Word game	Listen to the word, and spell the letters backward	7	Number of hits on the first attempt (7 points in total)
	Playground	Listen to the playground rides that the granddaughter wants to ride, and choose them in order	3	Number of hits on the first attempt (3 points in total)
Visuospatial function	Card game	Choose the same shape as what was presented	5	1 point for each hit
	Flower matching	Match pictures of the same flower	4	1 point for each hit + 1 point for hit on the first attempt
Executive function	Toilet use	Help the granddaughter with toilet use procedures	3	3-failure count
	Snack time	Helping in the process of granddaughter’s snack time	3	3-failure count

The VR test is designed to be conducted alone without the help of the staff; however, for subjects who are not familiar with VR, researchers in the treatment room helped in putting on the VR devices and initially implement them, while ensuring basic safety. At the start of the intervention, the participants stayed in the VR living room as long as they wanted to, and had some time to familiarize themselves with the device and also screen and practice clicking the letters to familiarize themselves with the controller. Afterward, the subjects performed the first part of the test with the virtual typical indoor house environment; and after a 10-min break, they performed the next part of the test with the virtual outdoor environment. The participants were notified in advance that they could immediately ask for help or stop if they experienced any feeling of discomfort, such as dizziness, nausea, or headache, during the test. All VR usage procedures and principles were applied equally in each center. The average use time of the VR test was 18.9 min, with the shortest use time being about 13 min and the longest time being about 32 min.

#### 2.2.4. Psychological assessment

Baseline psychological evaluation was conducted on the patients in the whole VARABOM project, and the results of the evaluation of depressive symptoms, emotional symptoms, and suicidal risk, which may affect cognitive function, were included in the analysis. In addition, the sickness which can be caused by VR use was evaluated after using the VR program.

##### 2.2.4.1. Brief screening for four mental illnesses of the elderly (BS4MI-Elderly)

The BS4MI-Elderly is a brief and comprehensive questionnaire designed to screen for important mental illnesses in the elderly (34). It covers four diseases: dementia, depressive disorder, sleep disorder, and hwa-byung, with high sensitivity (dementia, 0.61; depressive disorder, 0.88; sleep disorder, 0.85; hwa-byung, 0.94) and specificity (dementia, 0.91; depressive disorder, 0.93, hwa-byung, 0.84; sleep disorder, 0.84). With a total of 14 binary questionnaires, 12 included questions on the core symptoms of the four diseases, while two questions were asked on the course and duration of the diseases. The results were classified into three groups: “normal (green),” if there are no symptoms; “risk (yellow),” if the course or duration of disease is not enough; and “disorder (red),” if the severe symptoms were maintained for a sufficient period of time.

##### 2.2.4.2. Short-form of the Korean Geriatric Depression Scale (SGDS-K)

The 15-item SGDS-K was used to evaluate depression. It consists of binary questions, with a cutoff score of 8. A score of 8 or higher is thought to be highly depressed. In previous studies, sensitivity and specification were 0.94 and 0.73, respectively (35).

##### 2.2.4.3. Protective and Vulnerable Factors Battery Test (PROVE) – Depressive symptoms (DS)

PROVE-DS is a questionnaire that evaluates depressive symptoms among the battery tools for selection of depression and mental health protection (36). It consists of 15 items in total, with a 0–4 point Likert scale, which evaluates depression-related symptoms over the past 2 weeks. A high score indicates a high level of depressive symptoms.

##### 2.2.4.4. PROVE – Suicide risk (SR)

Six questions were based on a questionnaire about suicide accidents and suicide risk in PROVE-SR (36). The scores assigned to each item were different; and the higher the score, the higher the risk of suicide. The total score ranged from 0 to 20 points, with low risk of suicide being 4 or less, moderate risk being 5–7 points, and high risk being 8 points or more.

##### 2.2.4.5. Virtual Reality Sickness Questionnaire (VRSQ)

Virtual Reality Sickness Questionnaire (VRSQ) is a tool for assessing sickness symptoms related to oculomotor and disorientation, which are particularly relevant to VR use, among the symptoms assessed by the simulator sickness questionnaire (SSQ). This scale is derived from the original SSQ. It consisted of nine items for measuring VR sickness (37). It is 4-point self-reported questionnaire that includes oculomotor and disorientation subdomains. Each subscore is summed to a total, and then adjusted using the following formula: {(sum of oculomotor score)/12 * 100} + {(sum of disorientation score)/15 * 100}]/2. A higher score means higher sickness, and a cutoff has not been determined. The lowest score is 0 and the highest score is 100. This scale can be used for a comparison between people using the same VR software or symptom change in single person after VR use.

#### 2.2.5. Salivary DHEA analysis

##### 2.2.5.1. Salivary collection

Participants were instructed to collect at least 1 ml of saliva four times at 21:00, immediately after waking up, 30 min after waking up, and 1 h after waking up. Drinks or foods other than water were prohibited for 30 min before the collection of saliva. The collected saliva was centrifuged after two freeze-thaw cycles for mucin precipitation, and only the supernatant was stored for analysis at -70 degrees.

##### 2.2.5.2. DHEA measurement in saliva

A hormone assay system based on the liquid phased-double antibody method was used for the sophisticated analysis of salivary steroids. DHEA by Sigma-Aldrich Chemical Co. (St. Louis, MO, USA) was used as the standard. Steroids were dissolved in methanol (1.0 mg/ml) and then diluted with 0.1 M gelatin containing phosphate buffered saline (GPBS, pH = 7.0, containing 0.015 M NaN3 and 0.1% gelatin). Iodine125-labeled DHEA, a component of the DHEA RIA kit, was used for the assay. DHEA antiserum (Biogenesis, Oxford, UK) cross-reacted with 6.3% with 5a-andorstain-3beta, 17beta-diol, 1.3% with androstenedione, 0.1% with testosterone, and less than 0.05% with other related compounds. The volume of the standards added to the assay tubes was 0.1 ml. An equal volume of charcoal-stripped saliva was added to the standard tubes as well as to the sample tubes. The total radioactivity of 125I-DHEA per tube was approximately 20,000 cpm, and the antiserum was diluted to approximately 30–35%. Standards and samples were incubated overnight at 4°C, and then an anti-rabbit goat IgG-coated cellulose bead solution was added to the assay tubes. After incubating at room temperature for 30 min, the samples were centrifuged (1,500 × *g*) for 20 min. Radioactivity in the pellet was measured using a gamma counter (Cobra 5005, Perkin Elmer Life and Analytical Sciences, MA, USA). The DHEA levels for individual assays were calculated by interpolation from the standard curves using the RiaSmart software (PerkinElmer Life and Analytical Sciences, MA, USA). The morning DHEA value, which is the sum of DHEA immediately after waking up and 30 min, was called DHEA_sum_ and used for analysis.

### 2.3. Statistical analysis

For the comparison of demographic and clinical characteristics, a two-tailed *t*-test was conducted for age, SGDS-K, PROVE-DS, and PROVE-SR scores; and chi-square analysis was performed for sex, education year category, marital status, occupational status, depressive disorder history, BS4MI group, medication history, and computer and smartphone use experience. The differences in VR test score, MOCA score, DHEA_sum_, and VRSQ by group were analyzed using the analysis of covariance (ANCOVA), with age and current psychiatric and cognitive medication history as covariates. To further investigate the validity of each test, a receiver operating characteristics (ROC) curve analysis was performed to determine the sensitivity and specificity of the VR test and MOCA. Based on the ROC curves, the best cut-off score for discriminating between healthy control and MCI was also investigated based on the Youden J index. The correlations between MOCA and VR scores, each VR task and subdomain scores, VR subdomain scores and total score, and VR scores and DHEA were examined using partial correlation analysis with the same covariates as above. The covariates used for each analysis are summarized in Supplementary Table 1. All statistical analyses were conducted using IBM SPSS version 25 (IBM Corporation, Armonk, NY, USA).

## 3. Results

### 3.1. Demographic and clinical characteristics

The demographic and clinical characteristics of each group are shown in Table 1. No between-group differences in age, sex, education, marriage, or occupational status were observed. In addition, there were no significant differences in the proportion of subjects with a history of psychiatric disease, a history of depression, current cognitive and psychiatric medications, degree of depression (PROVE-DS), suicide risk (PROVE-SR), and risk of mental illness (BS4MI) between the two groups. Of the 10 people suspected of having mental health disorders in BS4MI in the normal group, six were still quite symptomatic among those with depression history, and four had no history of psychiatric diseases but currently had symptoms, such as insomnia or depression. Regarding digital literacy, participants in each group showed no differences in smartphone and computer use experience. In both groups, most of the subjects had an experience of using smartphones (normal 97.2%; MCI, 91.7%), and more than half of the participants had an experience of using computers (normal, 63%; MCI, 50%).

### 3.2. VR cognitive test and MOCA results by group

Table 3 shows the differences in the VR cognitive test and MOCA results for each group. The total MOCA score did not show differences between the MCI and normal groups, but the total score on the VR test displayed a significant difference (MOCA, mean of normal = 24.39, MCI = 22.83, *F*_1_,_1_ = 2.36, *p* = 0.127; VR mean of normal = 34.88, MCI = 27.67, *F*_1_,_1_ = 8.774, *p* = 0.004). As for the score by cognitive subdomains, the memory subdomain score differed in the VR test, and the attention and orientation subdomain scores differed in the MOCA test (VR memory, mean of normal = 18.83, MCI = 15.5, *F*_1_,_1_ = 17.301, *p* < 0.001; MOCA attention, mean of normal = 5.14, MCI = 4.42, *F*_1_,_1_ = 4.143, *p* < 0.001; MOCA orientation, mean of normal = 5.88, MCI = 5.42, *F*_1_,_1_ = 10.685, *p* = 0.001).

**TABLE 3:** Scores of VARABOM test and MOCA-K assessment by group.

	Normal (N = 108)	MCI (N = 12)	F	P	Partial eta squared
VARABOM test, mean (SD)					
Total score	34.88 (6.55)	27.67 (8.87)	8.674	0.004**	0.07
Memory	18.83 (2.24)	15.5 (3.99)	17.301	<0.001***	0.131
Attention	5.89 (3.76)	3.58 (4.23)	2	0.16	0.017
Executive function	3.78 (1.29)	3.08 (2.02)	1.914	0.169	0.016
Visuospatial	6.4 (1.72)	5.5 (1.93)	1.763	0.187	0.015
**MOCA-K, mean (SD)**					
Total score	24.39 (3.4)	22.83 (4.69)	2.36	0.127	0.02
Delayed recall	2.55 (1.61)	2.5 (1.73)	0.046	0.83	<0.001
Attention	5.14 (1.18)	4.42 (1.24)	4.143	0.044*	0.035
Executive function-visuospatial	4.06 (1.1)	3.92 (1.08)	0.323	0.571	0.003
Naming	2.92 (0.3)	2.75 (00.62)	2.238	0.137	0.019
Language	2.44 (0.63)	2.42 (0.67)	0.039	0.843	<0.001
Orientation	5.88 (0.38)	5.42 (0.9)	10.685	0.001**	0.085

VARABOM, virtual reality-based MCI monitoring; MOCA-K, Montreal Cognitive Assessment-Korean version; MCI, mild cognitive impairment; SD, standard deviation.

*p < 0.05; **p < 0.01; ***p < 0.001.

**FIGURE 2:**
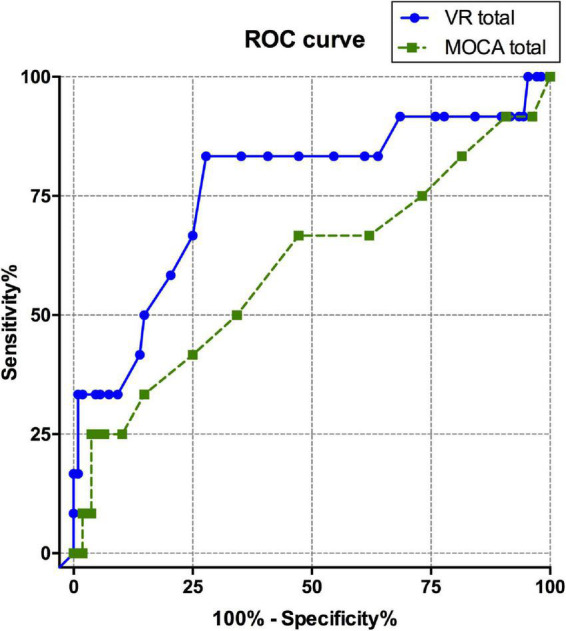
ROC curve comparison between total scores of VARABOM test and MOCA-K test. ROC curves with VR total score (blue-solid line) and MOCA total score (green-dashed line). ROC curve, receiver operating characteristic curve; VARABOM, virtual reality-based MCI monitoring; MOCA-K, Montreal Cognitive Assessment-Korean version.

The ROC curve was calculated to evaluate the diagnostic sensitivity and specificity of the VR cognitive and MOCA tests (Table 4 and Figure 2). The area under the curve (AUC) of the VR test was 0.765, and that of the MOCA test was 0.598, which was higher in the VR test. At the optimal cut-off according to the Youden’s J index, sensitivity was 0.833 for VR, 0.25 for MOCA, and the specificity was 0.722 for VR and 0.963 for MOCA.

**TABLE 4:** Statistical comparison between the ROC curves of VARABOM test and MOCA-K test.

Test	Sensitivity	Specificity	AUC	SE	95% C	*p*	Optimal cut-off value (Youden’s J Index)
VARABOM test	0.833	0.722	0.765	0.083	0.6–0.93	0.003	31.5 (0.555)
MOCA-K	0.25	0.963	0.598	0.098	0.41–0.79	0.265	17.5 (0.213)

ROC curve, receiver operating characteristic curve; VARABOM, virtual reality-based MCI monitoring; MOCA-K, Montreal Cognitive Assessment-Korean version; AUC, area under the curve; SE, standard error; CI, confidence interval.

### 3.3. Association of VR cognitive test and MOCA test

**FIGURE 3:**
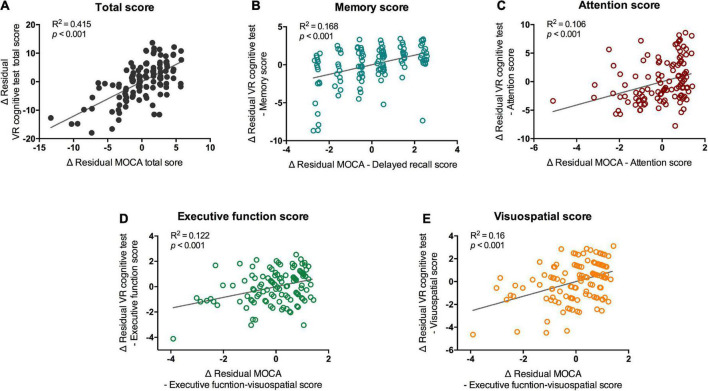
Partial correlation of scores of VR cognitive test and MOCA test. VR, virtual reality; MOCA-K, Montreal Cognitive Assessment-Korean version. Correlation between VR scores and MOCA scores. Correlation between **(A)** total scores, **(B)** memory scores **(C)** attention scores, **(D)** executive functions, and **(E)** visuospatial scores of MOCA and VR test were analyzed with partial correlation test. Line and *R*^2^ value indicate regression of the dataset.

The results of the VR cognitive and MOCA tests showed significant positive correlations between the total score and subdomains in the partial correlation analysis that controlled for age and medication (Figure 3A; total score, *R*^2^ = 0.415, *p* < 0.001). Memory, attention, executive function, and visuospatial function all showed significant correlations with the corresponding subdomains of the MOCA (Figures 3B–E; Memory, *R*^2^ = 0.168, *p* < 0.001; attention, *R*^2^ = 0.106, *p* < 0.001; executive function, *R*^2^ = 0.122, *p* < 0.001; visuospatial function, *R*^2^ = 0.16, *p* < 0.001). When in-group analysis was performed, the total score showed a significant correlation between VR and MOCA in both groups (normal group, *r* = 0.621, *p* < 0.001; MCI group, *r* = 0.763, *p* = 0.017), but correlations between subdomain scores were observed only in the normal group (Supplementary Table 2).

### 3.4. Subdomains of VR cognitive test

Looking at the correlation between each task category of the VR test and the score of each subdomain of the VR test, the correlation coefficient of the matching subdomain to which each task category of the VR test belongs was higher than those of the other subdomains (Table 5). In addition, the correlation between the subdomain score and the total score for each cognitive subdomain was higher than that of the other areas, indicating that the classification of each cognitive subdomain of this VR test is acceptable (Table 6).

**TABLE 5:** Correlation coefficients of each task of VARABOM test with total score and cognitive subdomains.

Task	Total score	Memory	Attention	Visuospatial function	Executive function
Preparation	0.548***	0.771***	0.229*	0.292**	0.25**
Preparation recall	0.468***	0.665***	0.248**	0.228*	0.089
Playground recall	0.248**	0.379***	0.059	0.134	0.201*
Road	0.322***	0.413***	0.238*	0.12	0.02
Hat finding	0.428***	0.517***	0.275**	0.215*	0.14
Phone call	0.516***	0.212*	0.581***	0.299**	0.193
Word game	0.724***	0.318***	0.891***	0.278**	0.169
Playground	0.401***	0.236*	0.479***	0.072	0.151
Card game	0.521***	0.474***	0.275**	0.652***	0.087
Flower matching	0.473***	0.162	0.246**	0.862***	0.265**
Toilet use	0.413***	0.283**	0.163	0.241**	0.778***
Snack time	0.263**	0.052	0.174	0.1	0.633***

VARABOM, virtual reality-based MCI monitoring.

*p < 0.05; **p < 0.01; ***p < 0.001.

**TABLE 6:** Correlation coefficients between each cognitive domain and total score of VARABOM test.

	Total score	Memory	Attention	Visuospatial function
Total score	–			
Memory	0.734***	–		
Attention	0.831***	0.377***	–	
Visuospatial function	0.633***	0.369***	0.331***	–
Executive function	0.485***	0.252**	0.236*	0.249**

VARABOM, virtual reality-based MCI monitoring. *p < 0.05; **p < 0.01; ***p < 0.001. The bold numbers represent the correlation coefficiency between the total score and other subdomain scores.

### 3.5. VR sickness score

**FIGURE 4:**
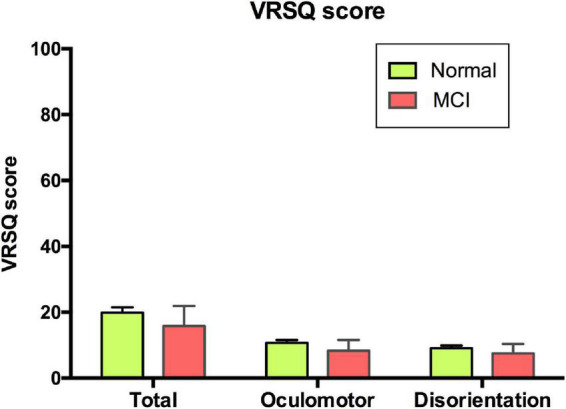
VRSQ total and sub-scores of each group. VRSQ, Virtual Reality Sickness Questionnaire; MCI, mild cognitive impairment.

The mean of total VRSQ score was 19.81 (SD 16.45) in the normal group and 15.83 (SD 21.14) in the MCI group. No differences in the VRSQ total scores as well as the oculomotor and disorientation scores were observed between the normal and MCI groups (total score, *F*_1_,_1_ = 0.886, *p* = 0.348; oculomotor score *F*_1_,_1_ = 0.959, *p* = 0.33; disorientation score, *F*_1_,_1_ = 0.651, *p* = 0.422; Figure 4).

### 3.6. Association of DHEA level with VR cognitive test

**FIGURE 5:**
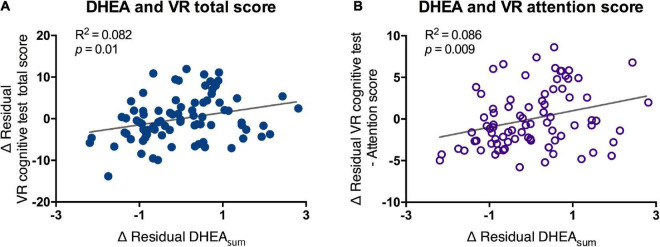
Partial correlation of scores of VR cognitive test and DHEA_sum_. VR, virtual reality; DHEA, dehydroepiandrosterone; AUC, area under the curve. Correlation between salivary DHEA and **(A)** VR total score and **(B)** VR attention score were analyzed by partial correlation test. Line and *R*^2^ value indicate regression of the dataset.

The morning DHEA level, which was obtained from the sum of DHEA (DHEA_sum_) in the period immediately after waking up and 30 min after waking up, showed a significant positive correlation with the total score and attention score of the VR test. The higher the VR total and attention scores, the higher the secretion of DHEA in the awakening phase (DHEA_sum_-total score, *R*^2^ = 0.082, *p* < 0.01; DHEA_sum_-attention score, *R*^2^ = 0.086, *p* < 0.009; Figure 5). However, no significant difference was found in the DHEA_sum_ between the MCI and normal groups (mean of normal = 5.53, MCI = 5.6).

## 4. Discussion

This study presents three main results. First, the VR cognitive assessment program was more sensitive than the existing screening tool for detecting cognitive impairment in MCI; and the score of this VR test was highly correlated with the existing MOCA test, not only by the total score, but also by the sub-domain scores. This demonstrates that the VR cognitive assessment program has the potential to become a screening tool for MCI. Second, the VR program was considered safe, as the participants did not report severe motion sickness or side effects after the VR tests. Finally, we found a significant correlation between the cognitive function and DHEA response during the awakening period, indicating that following research is warranted on this topic.

In this study, we attempted to explore the feasibility of a VR cognitive-assessment program in a real-life context in the older adults. We obtained data from the general older adult population and classified MCI groups based on the GDS score, which delineates the stages of cognitive and functional decline (26, 29). The group classified as MCI in this study may have been in the early stage of cognitive decline, since the MOCA total score between the normal and MCI groups did not show a significant difference. A recent study showed differences in VR scores only between MCI and normal groups, and no differences in MOCA, MMSE, and detailed neuropsychological battery scores (33). In other studies, MOCA is also known as a sensitive test for detecting early cognitive impairment; therefore, it is possible that the subjects in this study had milder cognitive impairment (30, 31). Nevertheless, the VR test in this study detected a minor decline with greater sensitivity among the MCI subgroups, with especially significant group differences for the memory subdomain, which is likely to be a useful tool for screening at the beginning of the pathway to AD. When a wider range of MCI patients are targeted, there is a risk that the specificity of the VR test may be relatively lower than that of the MOCA; however, VR test could be used as a screening test due to its sensitivity over specificity.

Our VR program was designed to evaluate various cognitive domains in a real-life context, unlike the existing VR cognitive test programs, which were evaluated mainly by spatial memory, episodic memory, or scoring scores with complex tasks without dividing the area (14). Compared to the other existing tests, which mainly used one or two tasks, 12 task types were performed in the same environmental context in this study, and the evaluation was conducted depending on the cognitive domains. Since the number of subjects in the MCI group was small, in group analysis within the subdomain scores of the MoCA and VR did not show significant correlations. However, in the whole group and the normal group, the total scores and the equivalent subdomain scores of MOCA and VR test were significantly correlated. Therefore, the subdomain classification in this test was thought to be valid. Previous studies have shown that VR tests evaluating multiple cognitive functions have higher sensitivity (13, 14, 38). According to the MCI and HC classification results from other studies, the VR reorientation test and VR path integration task, which measured the target or path-finding spatial memory, showed an AUC of 0.9 and 0.82, and sensitivity of 80.4 and 84%, respectively (16, 39). The virtual daily living test program, which measured the executive function using a daily living test, showed an AUC of 0.93 and sensitivity of 90% (19). In contrast, in the case of VR programs that measured various domains in combinations, the HC, MCI, and AD were detected at 100% prediction rate for all machine learning algorithms, indicating the usefulness of the multidomain test (38). The results of this study included lower scores, such as an AUC of 0.77 and sensitivity of 83.3%, which was most likely because the subjects in this study had minor cognitive deficits, supported by the low sensitivity of the MOCA scores at 0.25. A subsequent study with more subjects will help to ensure an accurate verification. These domain-specific evaluations can also be used to classify MCI subtypes, which will help patients customize cognitive rehabilitation programs for particularly vulnerable areas.

Regarding ecological validity, the included tasks were closely related to the daily lives of older adults compared to a majority of existing VR neuropsychological tests, and had a high level of verisimilitude. In a familiar home and exteriors of the house, the older adults can have a great sense of immersion and comfort while carrying out tasks such as packing common items, dialing phone numbers, and helping with snack time. A large number of the existing VR tests do not portray the daily lives of older adults, and instead, they give the impression of video games, including performing missions such as treasure hunting, finding a way, or escaping from a disaster (11, 16, 18). Although these programs have performed well in discrimination of MCI and normal group, the VARABOM test has the advantage of being universally accepted and comfortable in an everyday environment.

In this study, the total VRSQ scores were 19.91 (SD 16.45) in the normal group and 15.83 (SD 21.14) in the MCI group. These scores are not of concern based on prior studies, which reported mean values of 11.87–44.46 (40–42). Moreover, our test could be considered as well-tolerated, as none of the subjects dropped out of the test due to motion sickness. It is believed that the subjects did not need to move in the VR scene, which caused less discomfort in the older adults, who are relatively prone to motion sickness. In addition, there was no subject who stopped the VR test due to a misunderstanding of the operation method. Although approximately 40–50% of the subjects had no computer use experience, they did not report any difficulty in choosing answers through an indicator with a joystick in their hands, rather than using a mouse. Using a mouse or keyboard is unnatural and requires more experience; interfaces, such as joysticks and tough screens in our VR test, enabled direct interactions which lessened unnecessary errors. In addition, many of the other VR tests were semi-immersive products that did not use an HMD. This is a fully immersive program that creates a more ecologically-valid environment and provides a higher sense of presence with tolerable simulator sickness (43, 44).

Since the ultimate goal of this study was to create a device that assists in the diagnosis of MCI objectively and accurately, we also conducted a feasibility analysis of correlation of possible biomarkers. Regarding brain function, we found that a decrease in DHEA was related to cognitive decline (45). In this study, no difference in morning DHEA levels was observed between the normal and MCI groups. However, there was a positive correlation between the total and attention scores of the VR test and morning DHEA after adjusting for age, as well as cognitive and psychiatric medication history. DHEA has been previously administered for the purpose of cognitive enhancement (23). However, clinical studies have shown that the link between DHEA and cognitive impairment or dementia remains controversial (45). In the study by Valenti and Sanders, DHEA had a positive correlation with attention and working memory, and low levels of DHEA was shown to be a marker of cognitive decline (46, 47). A meta-analysis that analyzed the relationship between DHEA and dementia showed that DHEA-sulfate (DHEA-S) has potential as a diagnostic tool for AD, since their levels were lower in patients with AD (48). In these studies, DHEA was analyzed in the serum, plasma, and CSF. Although salivary DHEA levels are closely associated with free plasma-DHEA levels, few studies have analyzed the DHEA extracted from saliva for cognition analyses, as seen in our study (49, 50). In one study of salivary steroid hormones, low morning DHEA was associated with confusion, which is possibly associated with a decrease in attention. In addition, the present study analyzed DHEA instead of DHEA-S using the existing analysis method; however, it was found that the detection of DHEA-S has a higher association with AD (48). This might be due to long half-life and stability of DHEA-S, or the process of conversion from DHEA to DHEA-S may have been related to cognitive degradation (44). These points could be the reason why a difference in DHEA in each group was not detected in this study. Nevertheless, considering that DHEA was related to VR scores, which sensitively detected the cognitive decline, research on salivary DHEA in older adults with plasma DHEA and its sulphate form will be helpful.

Although we have demonstrated the possibility of using VR cognitive testing as an MCI screening tool, this study had several limitations. First, the present study screened the older adult community members with a wide range of inclusion criteria as a pilot study. Since it was not designed as a clinical trial for MCI diagnosis from the beginning, there were only 12 people in the MCI group, which is a very small number. Due to this small number of subjects in the disease group, we may not have been able to capture differences in some values, such as MOCA test scores. Additional studies should be performed to validate our VR test, in which experts properly diagnose MCI and recruit sufficient number of participants by group, age, and sex. Second, MCI could not be classified based on detailed neuropsychological tests, such as MMSE or IADL. Instead, we classified MCI on the basis of GDS, and the data that was collected within the cohort. In order to belong to GDS stage 3, the subjects must correspond to the Petersen Criteria, which is used as the current standard in MCI diagnosis (5). Therefore, the classification of MCI with GDS was simple but reasonable. Also, the validity of MCI classification was somewhat complemented since the GDS scoring was performed by trained psychiatrist and psychologist. Further clinical trial should be performed by recruiting MCI subjects prospectively and designing the inclusion criteria in a more sophisticated manner. Third, we did not control the depressive symptom scores or psychiatric diagnosis rates in this study, although these factors were not siginificantly different between the two groups. Fourth, this study has included not only drug-naïve subjects but also people taking neuropsychotropic drugs, such as Ginkgo biloba, choline alfoscerate, and antidepressants, which could influence the results. However, in the real world, several people are already on these drugs and are being tested for MCI diagnosis. Our study has an aspect that reflects real life, and we tried to control the confounding effect of the medication during statistical analysis with covariates. However, further studies should be conducted in drug-naive individuals for further scientific verification of our results. Fifth, since the comparative tool was limited to MOCA scores in our study, a more precise neuropsychological test should be included in future studies.

## 5. Conclusion

In conclusion, our VR cognitive test showed its potential as a screening software program for detecting minor cognitive decline in MCI with high sensitivity. Unlike the existing tools, it covered various cognitive domains and specified scores for each cognitive subdomain, indicating its potential to determine the patients’ MCI type and progression to dementia. Our findings on the relationship between salivary DHEA levels and cognitive function also suggest that it is worthy of further study as an MCI biomarker. A definitive study that divides groups more precisely and recruits sufficient number of subjects for each group will help to verify the validity of these screening tools.

## Data availability statement

The raw data supporting the conclusions of this article will be made available by the authors, without undue reservation.

## Ethics statement

The studies involving human participants were reviewed and approved by Institutional Review Boards of Severance Hospital, Institutional Review Boards of Gangnam Severance Hospital, Institutional Review Boards of Yongin Severance Hospital, and Institutional Review Boards of Ajou University. The patients/participants provided their written informed consent to participate in this study.

## Author contributions

J-HS, SJ, SS, JO, JH, WK, HR, KK, SL, and CH devised the project, main ideas, and provided proof of the outline. SS, HR, SJ, S-WC, and J-HS wrote the scenario and designed the VR program. HC and SK developed the VR program. S-WC, EJ, WC, JK, I-YK, J-YL, HS, and JR examined the participants and acquired and organized the data. RA conducted the salivary cortisol analysis. SJ drafted the manuscript, performed statistical analysis, and designed the figures. J-HS supervised this project. All authors discussed the results and commented on the manuscript.

## Supplementary material

Supplementary Video 1 Clip of “Preparation” mission.

Supplementary Video 2 Clip of “Phone call” mission.
