# Supplementary material for: Virtual reality-based monitoring test for MCI: A multicenter feasibility study
Source: Front Psychiatry. 2023 Jan 18;13:1057513. doi: 10.3389/fpsyt.2022.1057513 (PMC9891464; doi:10.3389/fpsyt.2022.1057513)
Supplement: Supplementary file 3 [file Table_1.DOCX]

**Supplementary Table 1. Covariate variables used in each analysis**

| Purpose of analysis | Analysis method | Covariate variables |
| --- | --- | --- |
| Comparison of age, SGDS-K, PROVE-DS and PROVE-SR scores by group | Two tailed t-test | None |
| Comparison of the ratio of sex, education status, marital status, occupational status, depressive disorder history, BS4MI group, medication history, computer and smartphone use experience by group | Chi-square analysis | None |
| Comparison of VR test score, MOCA score, DHEA_sum_ and VRSQ score by group | ANCOVA | Age, current psychiatric and cognitive medication |
| Investigate validity of VR and MOCA test to discriminate normal and MCI group | ROC curve analysis | None |
| Correlations between MOCA and VR scores | Partial correlation analysis | Age, current psychiatric and cognitive medication |
| Correlations between VR score and DHEA_sum_ | Partial correlation analysis | Age, current psychiatric and cognitive medication |

SGDS-K, short form of the Korean Geriatric Depression Scale; PROVE, Protective and Vulnerable factors battery questionnaire; DS, depressive symptomatology; SR, suicide risk; BS4MI-elderly, brief screening for four mental illnesses in the elderly; VR, virtual reality; MOCA, Montreal Cognitive Assessment; DHEA, dehydroepiandrosterone; VRSQ, Virtual Reality Sickness Questionnaire; MCI, mild cognitive impairment.

**Supplementary Table 2. Correlation coefficients between VR cognitive test and MOCA test by group**

|  | Whole participants  (n = 120) | Normal  (n = 108) | MCI  (n = 12) |
| --- | --- | --- | --- |
| VR total score & MOCA total score | 0.644^***^ | 0.621^***^ | 0.763^*^ |
| VR memory score & MOCA delayed recall score | 0.41^***^ | 0.406^***^ | 0.505 |
| VR attention score & MOCA attention score | 0.326^***^ | 0.295^**^ | 0.428 |
| VR visuospatial function score & MOCA executive-visuospatial function score | 0.4^***^ | 0.393^***^ | 0.47 |
| VR Executive function score & MOCA executive-visuospatial function score | 0.349^***^ | 0.329^**^ | 0.524 |

VR, virtual reality; MOCA, Montreal Cognitive Assessment; MCI, mild cognitive impairment. ^*^, p < 0.05; ^**^, p < 0.01; ^***^, p < 0.001.
